# Tracheal Aspirate and Bronchoalveolar Lavage as Potential Specimen Types for COVID-19 Testing Using the Cepheid Xpert Xpress SARS-CoV-2/Flu/RSV

**DOI:** 10.1128/spectrum.00399-22

**Published:** 2022-05-18

**Authors:** Sang Ho Kim, Fatimah AlMutawa

**Affiliations:** a Schulich School of Medicine and Dentistry, Western University, London, Ontario, Canada; b Department of Pathology and Laboratory Medicine, Western University, London, Ontario, Canada; c London Health Science Center, London, Ontario, Canada; University of Arizona/Banner Health

**Keywords:** SARS-CoV-2, multiplex, lower respiratory tract specimen, COVID-19, SARS-CoV-2, Xpert Xpress SARS-CoV-2 assay

## Abstract

Xpert Xpress SARS-CoV-2/Flu/RSV is a rapid diagnostic test currently approved for the detection of SARS-CoV-2 using upper respiratory tract specimens. This study attempts to assess the performance of this assay using upper and lower respiratory tract specimens by comparing its results to the lab-developed PCR test. We assessed the performance of GeneXpert for the detection of SARS-CoV-2, influenza A, influenza B, and respiratory syncytial virus for upper respiratory tract specimens. In addition, the SARS-CoV-2 detection was evaluated for lower respiratory tract specimens (bronchoalveolar lavage and tracheal aspirate). Precision and reproducibility of the test were also assessed using samples with varying cycle threshold values. Xpert Xpress SARS-CoV-2/Flu/RSV shows 100% positive and negative agreements for all four targets when tested using upper respiratory tract specimens. For lower respiratory tract specimens, tracheal aspirate and bronchoalveolar lavage samples respectively show 96% and 100% positive percent agreement for SARS-Cov-2 target only. No positive flu/RSV samples were included for lower respiratory tract specimens. Both samples show 100% negative percent agreement. The precision and reproducibility assay also showed 100% correspondence. Xpert Xpress SARS-CoV-2/Flu/RSV can be potentially used for SARS-Cov-2 detection in lower respiratory tract specimens. Performance passed our study acceptance criteria and shows promising implications as a point of care detection assay.

**IMPORTANCE** Cepheid’s Xpert Xpress SARS-CoV-2/Flu/RSV provides a means of rapid diagnosis that can help in hospital bed management and patient flow. It is also important for each microbiology lab to increase its capacity and most importantly have a different platform to overcome the anticipated reagent shortage at times of peak community transmission. There is limited evidence on using it for lower respiratory tract specimens. Here we present our validation for upper respiratory tract specimens as well as a potential use for lower respiratory specimens (BAL and TA), and we discuss some of the applications we have been using in our organization.

## INTRODUCTION

The continuation of the severe acute respiratory syndrome coronavirus 2 (SARS-CoV-2) pandemic has called for a need for accurate and rapid diagnostic tests to minimize the spread of the diseases. In response to this demand, the U.S. Food and Drug Administration (FDA) has approved several rapid tests as a tool for the detection of SARS-CoV-2 under emergency use authorization (EUA) (1). One of these tests is an Xpert Xpress SARS-CoV-2/Flu/RSV. Xpert Xpress SARS-CoV-2/Flu/RSV is a rapid, real-time RT-PCR test for the qualitative detection and differentiation of SARS-CoV-2, influenza A, influenza B, and respiratory syncytial virus (RSV) (1). The test’s portability, short turnaround time, and ability to simultaneously detect multiple infections make it a suitable point of care detection method during these times. Currently, Health Canada and the FDA have approved Xpert Xpress SARS-CoV-2/Flu/RSV as a point of care detection only for upper respiratory tract (URT) swab specimens (1). However, lower respiratory tract (LRT) swab specimens, including tracheal aspirate (TA) and bronchoalveolar lavage (BAL), have shown promising implications (2). The present study aimed to assess the performance of Xpert Xpress SARS-CoV-2/Flu/RSV by comparing the test results to the lab-developed test (LDT) of reverse transcriptase-PCR (RT‐PCR). For URT swab specimens, we evaluated the assay for the detection of SARS-CoV-2, influenza A and B, and RSV. For LRT swab specimens, only SARS-CoV-2 results were validated.

## RESULTS

Xpert Xpress SARS-CoV-2/Flu/RSV assay using URT demonstrated a strong agreement with the LDT. The two tests were in 100% agreement for the four analytes included in the study when using specimens collected from the upper respiratory tract (Table 1).

**TABLE 1:** Percent agreement between Xpert Xpress SARS-CoV-2/Flu/RSV and the lab-developed PCR method for detecting different analytes using URT specimens^*a*^

		LDT			
Xpert Xpress		Positive	Negative	PPA (±95% CI)	NPA (±95% CI)
SARS-CoV-2	Positive	21	0	100% (0.74–1)	100% (0.91–1)
	Negative	0	40		
Influenza A	Positive	10	0	100% (0.69–1)	100% (0.91–1)
	Negative	0	51		
Influenza B	Positive	10	0	100% (0.69–1)	100% (0.91–1)
	Negative	0	51		
RSV	Positive	10	0	100% (0.69–1)	100% (0.91–1)
	Negative	0	51		

a *n* = 61 for each target.

TA and BAL specimens were analyzed as LRT samples. Positive agreements between Xpert Xpress SARS-CoV-2/Flu/RSV assay and the LDT were 96% and 100% for TA and BAL samples, respectively. Both samples had a negative percent agreement of 100% (Table 2).

**TABLE 2:** Percent agreement between Xpert Xpress SARS-CoV-2 and the lab-developed PCR method for the detection of different analytes using LRT specimens: TA and BAL

		LDT			
Sample Type	Xpert Xpress	Positive	Negative	PPA (±95% CI)	NPA (±95% CI)
Tracheal	Positive	24	0	96.0% (0.80–1)	100% (0.75–1)
Aspirate	Negative	1	13		
Bronchoalveolar	Positive	16	0	100% (0.79–1)	100% (0.80–1)
Lavage	Negative	0	17		

The first precision assay using varying Ct values showed 100% agreement for all concentrations. The second analysis using in-house control (1) also showed 100% correspondence with a 1.5% coefficient of variation. The reproducibility test showed consistently positive results for all 10 tested replicates (Ct value: 37–39). Both precision and reproducibility were within the study’s acceptance criteria. In addition, 40 URT samples with Ct >40 were also run and only 29 were detected (72%) (Table 3), estimating the limit of detection to be Ct less than 40.

**TABLE 3:** Limit of detection approximation using *Xpert Xpress SARS-CoV-2* for lower respiratory samples

Ct range	No. of samples detected/no. of samples tested	Detection percentage
37–39	10/10	100%
41–42	18/24	75%
43–44	11/16	68.75%

Analytical specificity was not conducted due to limited kits caused by supply shortages during the pandemic. There were a few samples that had other respiratory pathogens including Streptococcus pneumoniae, Haemophilus influenzae, alpha-hemolytic streptococci, and coagulase-negative staphylococci. These pathogens did not interfere with the test results. This indicates no evidence of cross-reactivity, but no conclusion was able to be drawn due to the limited numbers.

## DISCUSSION

Similar to other studies utilizing URT specimens (2–4), our results showed a strong positive and negative percent agreement between Xpert Xpress SARS-CoV-2/Flu/RSV assay and the LDT for all analytes. For LRT specimens, a study by Wong et al. showed 100% positive and negative agreements with respect to their lab-developed PCR test (2). This corresponds to our results, which also showed strong positive and negative agreements using these specimens. The one sample that provided discordant results between Xpert Xpress SARS-CoV-2/Flu/RSV and LDT had a Ct value of 31.7. Due to sample depletion, no further testing was done. In our study, specimens collected from bronchoalveolar lavage demonstrated a slightly greater agreement compared to those from tracheal aspirate (1.00 vs.0.96). In addition, our cutoff for reporting positive was Ct <40. Any specimen with Ct >40 gave inconsistent results, implying that these samples may represent false positives, a previously infected patient who is still shedding, or early infection.

Xpert Xpress assay delivers results in a relatively short turnaround time of a maximum of 45 min. The test also integrates sample preparation, nucleic acid extraction, RT-PCR amplification, and sequence detection into a single cartridge, minimizing cross-contamination between samples. The single-cartridge method also offers flexibility as each sample can be run at different times whenever prepared, unlike the PCR method which requires all samples to run simultaneously. On the other hand, the test has a low throughput rate compared to the LDT, which can load hundreds of samples at once. It also costs approximately $89 CAD (Canadian dollar) per kit, which is much more expensive than the LDT, which costs around $11 CAD per kit. Despite the limitations, the ability of the assay to deliver rapid and accurate results is necessary for controlling the spread of the disease. A study by Zhen et al. demonstrates that Xpert Xpress SARS-CoV-2/Flu/RSV also shows a stronger analytical and clinical sensitivity compared to other point-of-care detection methods like Abbott ID NOW and GenMark ePlex (5).

One of the clinical applications of Xpert Xpress SARS-CoV-2/Flu/RSV is in bed management in hospitals, which is critical, especially with the third wave of the pandemic. Unlike the LDT, which only allows batch testing, the rapid test has the flexibility of not having to run samples in batches. With faster turnaround time and around-the-clock service, the rapid test allows a more average number of samples to be processed daily (6). This can alleviate the emergency room demands by increasing the bed turnover rate for new admissions. Such efficiency is also necessary for critical care patients where rapid results are required for managing safe environments.

As mentioned above, Health Canada and the FDA have allowed the Xpert Xpress SARS-CoV-2/Flu/RSV assay to be utilized as a point of care detection device for URT specimens in Canada and the U.S. (1). The strong agreements in our results using LRTs indicate that the assay can expand its use to these specimens as well. This is helpful for patients with lung transplants or those in an intensive-care unit on mechanical ventilation. Additionally, LRT samples also have been shown to provide better diagnostic values than URT specimens for patients with severe acute respiratory infection caused by SARS-CoV-2 (7).

The study comes with some limitations. The disrupted supply chain from the pandemic restricted supplies for the study, hindering the extensivity of the validation as initially planned. For instance, the limit of detection was only roughly estimated, and analytical specificity was not able to be conducted. Cross-reactivity was another study that could not be completed. The manufacturer evaluated this using *in silico* analysis using multiple strains with no cross-reactivity seen (8). There is also no information regarding patient demographics as secondary sample validation was done on previously collected samples and tested anonymously. With regard to a limitation to the assay itself, the FDA indicated that the sensitivity of detecting the N2 target may be affected by a single point mutation, but the E gene will still be detected, leading to presumptive results since the assay is designed to detect multiple targets (9). This reinforces the importance of correlating those results clinically and being aware of circulating variants that may affect the instrument interpretation. It is crucial for the lab to develop a guide on how to report those results and communicate to the clinical team where patients are reassessed and tested again if clinically indicated. Cepheid has recently released Xpert Xpress CoV-2/Flu/RSV plus, which now has three distinct gene targets: N2, E, and RdRP (10). Increasing the number of targets could improve the variant detection and mitigate the effects of viral mutations.

To conclude, we assessed the performance of Xpert Xpress SARS-CoV 2/Flu/RSV using upper and lower respiratory tract specimens. URT specimens were tested for agreement with SARS-CoV-2, influenza A, influenza B, and RSV, while LRT specimens were tested only for SARS-CoV-2. The results indicate that the assay meets the 90% acceptance criteria in comparison with the reference PCR method. Availability and appropriate assessments of rapid tests are vital in controlling the spread of the current pandemic. With its accuracy and rapidness, Xpert Xpress SARS-CoV-2/Flu/RSV can provide meaningful results, and this use may be extendable to LRT specimens. Future studies are needed on further validation, including a complete limit of detection and analytical specificity, and looking at the implications of using LRT samples to detect SARS-CoV-2 using Xpert Xpress SARS-CoV-2/Flu/RSV, which can reveal the potential benefits of utilizing these specimens.

## MATERIALS AND METHODS

The assay was validated using two types of specimens: upper and lower respiratory tract specimens. Sixty-one URT samples were tested for the four targets: SARS-CoV-2, influenza A and B, and RSV. For SARS-CoV-2, we tested 21 positive and 40 negative samples. For all other analytes, we tested 10 positive and 51 negative samples. For LRT samples, 38 TA samples and 33 BAL samples were included in the study and evaluated for SARS-CoV-2 targets only. The other targets have not been evaluated for LRT specimens.

Spiked negative samples and patient samples with known analytes were used for this study. The majority of LRT samples were prepared by spiking, while all URT samples were from previously tested patients. The upper respiratory samples were collected with a single swab and transported in a universal transport medium (UTM). All collections were done using a nasopharyngeal swab except for two samples that used nasal swabs. For lower respiratory samples, spiked known negative specimens were prepared by adding Dithiothreitol (100 μL of 65 mM Dithiothreitol in PBS + 400 μL water) at a 1:1 ratio for homogenization to a 1 mL lower respiratory specimen. The addition of Dithiothreitol did not affect the detection. Fifty μL of the SARS-CoV-2 sample was then added. The analytes used for spiking were clinical samples that were tested and confirmed previously in our lab. Those samples were collected in a sterile container and kept frozen at −80.

Samples were subjected to heat inactivation at 56° for 30 min for safety purposes. This was done as part of the laboratory routine testing for the LDT PCR. This did not affect the results of the assay during our study. Three hundred L of samples was used for testing.

Accuracy was assessed by examining the positive and negative percent agreement of different analytes. Precision was analyzed through two experiments using URT specimens. First, previously tested samples were run at varying Ct values: strong positive (Ct 15), moderate positive (Ct 22), and negative specimen. All runs were done in replicates on both instruments by different users in different shifts (1). The second precision assay was done by running in-house controls with all four targets in replicates (2 on each instrument for 5 days) for a total of 20 using 2 different lot numbers. The precision was assessed by calculating the coefficient variation of Ct values. The reproducibility was evaluated by testing 10 replicates of weak positive samples (Ct value between 37 and 39) using LRT specimens. We also tested LRT specimens with Ct value >40 in replicates for a total of 40 replicates to roughly estimate our limit of detection. For accuracy, agreement over 90% was considered passing the acceptance criteria. The acceptance criteria for reproducibility and precision were 95% and coefficient variation <5%.

For the LDT, a research-use-only E-gene/EAV assay (cat no. 40-0776-96, TIB MOLBIOL) and RNA Virus Master (cat no. 06754155001, Roche) were used. The extraction was done by Hamilton Liquid Handler and Promega HT TNA, followed by an analysis using LightCycler 480 II instruments. Ten μL of master mix and 10 μL of eluate were used in every reaction. For validation, the Xpert Xpress SARS-CoV2/Flu/RSV kit was used using the GX-XVI - 16 module instrument with a desktop computer (Cat No. GXXVI-16-D, Cepheid).

For statistical analysis, the positive and negative agreements and their respective confidence interval (CI) were calculated using Microsoft Office Excel 365 (Microsoft, Redmond, WA).
